# miR-221 affects multiple cancer pathways by modulating the level of hundreds messenger RNAs

**DOI:** 10.3389/fgene.2013.00064

**Published:** 2013-04-25

**Authors:** Laura Lupini, Cristian Bassi, Manuela Ferracin, Nenad Bartonicek, Lucilla D'Abundo, Barbara Zagatti, Elisa Callegari, Gentian Musa, Farzaneh Moshiri, Laura Gramantieri, Fernando J. Corrales, Anton J. Enright, Silvia Sabbioni, Massimo Negrini

**Affiliations:** ^1^Dipartimento di Morfologia, Chirurgia e Medicina Sperimentale, Università di FerraraFerrara, Italy; ^2^Laboratorio per Tecnologie delle Terapie Avanzate, Tecnopolo, Università di FerraraItaly; ^3^EMBL - European Bioinformatics Institute Wellcome Trust Genome CampusHinxton, Cambridge, UK; ^4^Department of Molecular Medicine, School of Advanced Technologies in Medicine, Tehran University of Medical SciencesTehran, Iran; ^5^Centro di Ricerca Biomedica Applicata, Policlinico S. Orsola-Malpighi e Universita di BolognaBologna, Italy; ^6^Center for Applied Medical Research, University of NavarraPamplona, Spain; ^7^Dipartimento di Scienze della Vita e Biotecnologie, Università di FerraraFerrara, Italy

**Keywords:** microRNA, miR-221, microarray, Sylamer, gene targets

## Abstract

microRNA miR-221 is frequently over-expressed in a variety of human neoplasms. Aim of this study was to identify new miR-221 gene targets to improve our understanding on the molecular tumor-promoting mechanisms affected by miR-221. Gene expression profiling of miR-221-transfected-SNU-398 cells was analyzed by the Sylamer algorithm to verify the enrichment of miR-221 targets among down-modulated genes. This analysis revealed that enforced expression of miR-221 in SNU-398 cells caused the down-regulation of 602 mRNAs carrying sequences homologous to miR-221 seed sequence within their 3′UTRs. Pathways analysis performed on these genes revealed their prominent involvement in cell proliferation and apoptosis. Activation of E2F, MYC, NFkB, and β-catenin pathways was experimentally proven. Some of the new miR-221 target genes, including RB1, WEE1 (cell cycle inhibitors), APAF1 (pro-apoptotic), ANXA1, CTCF (transcriptional repressor), were individually validated as miR-221 targets in SNU-398, HepG2, and HEK293 cell lines. By identifying a large set of miR-221 gene targets, this study improves our knowledge about miR-221 molecular mechanisms involved in tumorigenesis. The modulation of mRNA level of 602 genes confirms the ability of miR-221 to promote cancer by affecting multiple oncogenic pathways.

## Introduction

miR-221 is among the most frequently up-regulated miRNAs in human cancer. It is over-expressed in a large fraction of glioblastoma, liver, bladder, thyroid, pancreatic, gastric, and prostate carcinomas (Gottardo et al., 2007; Lee et al., 2007; Visone et al., 2007; Fornari et al., 2008; Mercatelli et al., 2008; Pineau et al., 2010; Liu et al., 2012; Quintavalle et al., 2012). In few cases, non-oncogenic functions of miR-221 were reported. For example, the regulation of c-kit by both miR-221 and miR-222 induced anti-angiogenic effects and the reduction of cell proliferation of herythroleukemic cells (Felli et al., 2005), suggesting that miR-221 effects could also depend on cellular context. Its suggested role in tumorigenesis was confirmed by the finding that miR-221 over-expression correlates with tumor aggressive features, such as the presence of metastasis and multifocal lesions in hepatocellular carcinoma (HCC) (Gramantieri et al., 2009; Fu et al., 2011). *In vitro* studies showed that miR-221 caused an increase in cell proliferation rate and invasion capability, while anti-miR-221 induced a decrease in cell growth and promoted apoptosis (Fornari et al., 2008; Garofalo et al., 2009; Pineau et al., 2010; Zhang et al., 2010b). *In vivo* studies proved that miR-221 could induce proliferation of tumorigenic murine hepatic progenitors cells (Pineau et al., 2010) and accelerate liver tumor formation in a miR-221 over-expressing transgenic mouse model (Callegari et al., 2012).

These observations underline the importance of miR-221 in tumorigenesis, in particular for HCC, and the value of understanding the molecular mechanisms controlled by miR-221. To this purpose, identification of gene targets could provide the links between miR-221 over-expression and pathways involved in human cancer. Some important gene targets have been already identified: the cyclin-dependent kinase inhibitors CDKN1B/p27 and CDKN1C/p57 (Galardi et al., 2007; le Sage et al., 2007; Fornari et al., 2008; Medina et al., 2008), the pro-apoptotic factors BMF (Gramantieri et al., 2009) and BBC3/PUMA (Zhang et al., 2010a,b), PTEN (Garofalo et al., 2009), an inhibitor of PI3K/AKT pathway, PTPμ (Quintavalle et al., 2012), a tyrosin-phosphatase that participates in cell adhesion regulation, and TIMP3 (Garofalo et al., 2009), a metallopeptidase inhibitor.

These miR-221 targets revealed tumor-promoting mechanisms associated with miR-221 over-expression. However, our knowledge about miR-221 molecular targets is largely incomplete and aim of this work was to reveal the many additional miR-221 targets with a high-throughput approach to identify most of its gene targets and improve our understanding of the multiple mechanisms that are affected by miR-221.

## Materials and methods

### Sylamer analysis and bioinformatics

Gene expression analysis of miR-221 transfected SNU-398 cells and Negative Control 2 (NC2, AM17111, Ambion) treated SNU-398 samples were performed using Agilent Whole Human Genome Oligo Microarray platform (Agilent Technologies), following manufacturer's procedures, as previously described (Ferracin et al., 2008). GeneSpring GX 11 software (Agilent Technologies) was used to analyze results. Data transformation was applied to set all the negative raw values to 1.0, followed by a quantile normalization. A filter on low gene expression was used to keep only the probes classified as Detected in at least one sample by the software. Array results were submitted in ArrayExpress (http://www.ebi.ac.uk/arrayexpress/, accession number E-MTAB-1531). Genes were ordered according to fold-change, from the most down-regulated to the most up-regulated in miR-221 transfected cells and the ordered gene list was analyzed with the Sylamer algorithm (EMBL-EBI), through the web-interface SylArray (http://www.ebi.ac.uk/enright-srv/sylarray) (Bartonicek and Enright, 2010). The Sylarray system assigned 3′UTR sequences to each mappable probe and filtered for low-complexity sequences, redundancy and multiple probe mappings. The Sylamer algorithm was applied to search for enrichment or depletion of miRNA seed sequences in the 3′UTRs ranked according to the gene list provided. The software generated plots representing the hypergeometric statistic significance of each nucleotide words across whole gene list. According to Sylamer method, the peak of the 7-mer plot closest to the start of the ranking was chosen as a conservative threshold to select putative miR-221 target genes (van Dongen et al., 2008). Algorithms used to identify predicted miR-221 target genes were TargetScan v. 5.2 (http://www.targetscan.org) (Lewis et al., 2005), MicroCosm v. 5 (http://www.ebi.ac.uk/enright-srv/microcosm/htdocs/targets/v5), Diana MicroT v.3 (http://diana.cslab.ece.ntua.gr/microT/) (Maragkakis et al., 2009). For pathway analysis we used GeneSpring GX v.11 and GeneGo (Nikolsky et al., 2005) software through the functions for finding significant pathways.

### Plasmid vectors

pMIF-GFP-miR-221 was prepared by cloning hsa-miR-221 gene in the pMIF-GFP-Zeo plasmid (System Biosciences), using the *Nhe*I restriction site. psiCheck-3′UTR constructs were prepared by cloning portions of 3′UTR (that contained miR-221 seed complementary regions) of putative target genes into psiCheck-2 vector (Promega), downstream of the renilla luciferase gene, using *Xho*I and *Pme*I restriction sites. The primers used to amplify 3′UTR regions and the lengths of cloned regions are listed in Table S1. psiCheck-RB1-3′UTR-mutated was obtained by deleting two 6-nucleotides regions, corresponding to miR-221 complementary regions, in RB1-3′UTR sequence of psiCheck-RB1-3′UTR. Mutagenesis was performed by Genescript company. All constructs were verified by sequencing.

### Cell cultures, transfections, and luciferase activity assays

SNU-398, HepG2, and Hek293, from American Type Culture Collection, were cultured in Iscove Modified Dulbecco's Medium (IMDM) supplemented with 10% fetal calf serum (Sigma-Aldrich) and 0.1% Gentamicin (Sigma-Aldrich).

For miRNA precursor transfection, cells were seeded in 24-well plates at a density of 100,000 cells/well, 24 h before transfection. Transfection was performed according to Lipofectamine2000 protocol (Invitrogen), using Optimem medium (Invitrogen-Gibco). *miR-221* precursor (AM17100—PM10337) and negative control #2 (NC2—AM17111) were obtained from Ambion and were transfected at the final concentration of 100 nM. The anti-miR-221 oligonucleotide was synthetized by Integrated DNA Technology (IDT) and it has the following sequence: 5′-mG^*^mA^*^mA mAmCmC mCmAmG mCmAmG mAmCmA mAmUmG mU^*^mA^*^mG^*^ mC^*^mU-3′ (m, methylated nucleotide; ^*^, phosphothioate bond). It was transfected at the final concentration of 100 nM.

Luciferase vectors (psiCheck-based vectors) were transfected at the final concentration of 800 ng/ml. Each transfection was performed in triplicate. For RNA extraction, cells were collected 48 h after transfection and the RNA was extracted following Trizol protocol (Invitrogen). For luciferase assays, firefly and renilla luciferase activity were measured using the Dual-Luciferase Reporter Assay (Promega), 24 h after transfection. The firefly luciferase activity was used to normalize the reporter renilla luciferase signal.

To experimentally investigate the involvement of miR-221 in cancer processes, we used the Cignal Finder 10-Pathway Reporter Arrays kit (SABiosciences), a commercial reporter array that allows for simultaneous evaluation of 10 cancer-related signaling pathways activation. It is a reverse transfection system that makes use of specific pathway-focused transcription factor-responsive firefly luciferase vectors. Ten thousand cells were seeded in wells containing the responsive and normalization vectors and luciferase activities were measured after 48 h. Data normalization was based on an included vector that expresses the renilla luciferase gene under the control of the strong cytomegalovirus (CMV) promoter.

### miR-221 stable cell clones

SNU-398 cells were transfected with pMIF-GFP-miR-221 plasmid. After 24 h from transfection cells were diluted and cultured in IMDM Medium supplemented with 10% fetal calf serum (Sigma-Aldrich), 0.1% Gentamycin (Sigma-Aldrich) and 400 μg/ml of zeocin. After 4 weeks, single colonies were picked up and miR-221 expression was evaluated using Real Time PCR.

### Real time PCR

The RNA purification by Trizol was performed according to manufacturer's indications (Invitrogen). For mature microRNA quantification we performed a Taqman Real time PCR, using *miR-221* probe (Applied Biosystems). Five nanogram of purified RNA were retro-transcribed using TaqMan MicroRNA Reverse Transcription kit (Applied Biosystems) and mature miR-221 MicroRNA Assay (Applied Biosystems, assays ID000524), following manufacturer's protocol. Real Time quantitative PCR was performed using TaqMan MicroRNA Assay specific for hsa-miR-221 (Applied Biosystems, assays ID000524) and for hsa-miR-222 (Applied Biosystems, assays ID002276). The reaction was carried out in a 96-well PCR plate at 95°C for 10 min followed by 40 cycles of 95°C for 15 s and 60°C for 1 min on Biorad-Chromo4 thermal cycler real-time PCR instrument. Each sample was analyzed in triplicate. The level of miRNA was measured using Ct (threshold cycle) and the amount of target was calculated using 2^−ΔΔCt^ method. To normalize the expression levels of miR-221, TaqMan endogenous control RNU6B (Applied Biosystem, assay ID001093) was used. For gene expression analysis we performed Real Time EvaGreen PCR. Five hundred nanogram of total RNA were retro-transcribed using random examers and oligo dT. Diluted cDNAs (1:5 for ANXA1, 1:10,000 for 18S and 1:100 for RB1, APAF1, WEE1, CTCF, GAPDH) were amplified in Real Time PCR using Qiagen Taq DNA Polymerase (QIAGEN, 201203) for EvaGreen (Biotium Inc.; 0.2× final concentration) detection. The following primers were used: RB1_2612F (TCA GAA GGT CTG CCA ACA CCA ACA), RB1_2744R (TGA GCA CAC GGT CGC TGT TAC ATA); APAF1_1046F (GGC TGT GGG AAG TCT GTA TTA G), APAF1_1195R (CAA CCG TGT GCA AAG ATT CTG); ANXA1_835F (TGG AGT TGA AAG GTG ACA TTG), ANXA1_982R (CGG GAA ACC ATA ATC CTG ATC); WEE1_1994F (CGA ATA GAA TTG AAT GCC GAA AAG), WEE1_2142R (GAT GTT CTA TTA CTC TGG GTG G); CTCF_1499F (AGT GTT CCA TGT GCG ATT AC), CTCF_1654R (GGG TTC TCA TGT GCC TTT TC); GAPDH_1107F (CTA TAA ATT GAG CCC GCA GCC), GAPDH_1257R (CCC AAT ACG ACC AAA TCC GT); 18S_616F (AGC AGC CGC GGT AAT TCC AGC T), 18S_784R (CGG GAC ACT CAG CTA AGA GCA TC). The reactions were incubated in a 96-well PCR plate at 95°C for 15 min followed by 40 cycles of 95°C for 30 s and 58°C for 30 s. Each sample was analyzed in triplicate. Fluorescence measurements were completed using a Biorad-Chromo4 thermal cycler real-time PCR instrument. The level of each mRNA was measured using Ct (threshold cycle) and the amount of target was calculated using 2^−ΔΔCt^ method. Gene expression levels were normalized using either 18S or GAPDH expression.

### Western blotting and antibodies

Cells were collected by trypsin-EDTA and dissolved in RIPA buffer (Radio-Immunoprecipitation Assay) (Sigma-Aldrich), supplemented with Protease inhibitors (Sigma-Aldrich), according to Manufacturer's protocol. The following antibodies were used: anti-human Retinoblastoma Protein (554136—BD Biosciences, 1:250), anti-APAF1 (clone E38, Millipore, 1:1000), anti-ANXA1 (sc-11387, Santa Cruz Biotechnology, 1:500), anti-WEE1, (sc-325, Santa Cruz Biotechnology, 1:500), monoclonal anti-actin (A4700, Sigma-Aldrich, 1:1000), HRP-linked anti-mouse antibody (A9044, Sigma-Aldrich, 1:20,000), HRP-linked anti-rabbit antibody (7074, Cell Signaling, 1:40,000). For signal detection LiteAblot Turbo Extra-Sensitive Chemiluminescent Substrate (Euroclone) was used. Signals were quantified by the ImageJ software (http://rsbweb.nih.gov/ij/) and protein expression levels were normalized according to β-actin expression.

### Statistical analysis

The significance of differential expression in luciferase assays and quantitative PCRs between groups was assessed by student's *t*-test.

## Results

### miR-221 can down-modulate hundreds of gene targets by reducing their RNA levels

To identify genes controlled by miR-221, we compared gene expression profiling of SNU-398 cells transfected with miR-221 precursor vs. SNU-398 cells transfected with negative control (NC2, Ambion). This cell line was chosen because it expresses low level of miR-221 (Fornari et al., 2008). In our experimental setting, miR-221 transient transfection induced a ≈10-fold increase in microRNA expression. No expression changes were detected for the related miR-222 (Figure S1). Three independent experiments of miR-221 transfections vs. four independent experiments of NC2 transfections were compared. Following quantile normalization and exclusion of undetectable or compromised probes, 26,586 *DETECTED* probes, according to Agilent Feature Extraction analysis software, were used.

**Figure 1:**
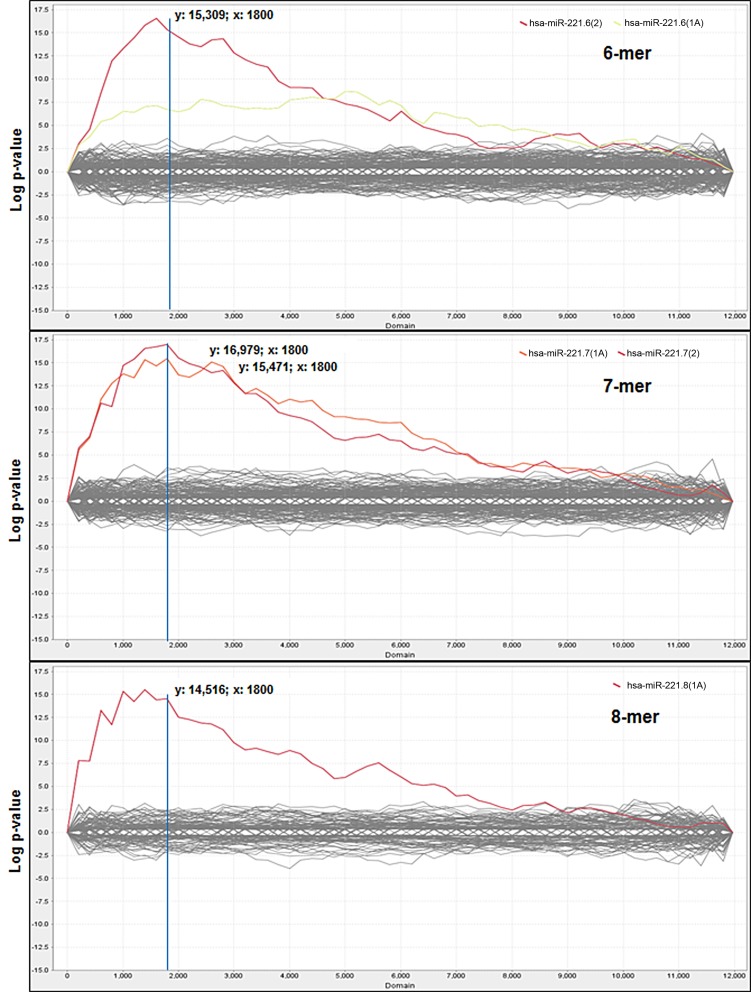
**Sylamer enrichment landscape plots.** Plots show the 6nt, 7nt, and 8nt seed complementary enrichment profiles. The graph shows that the words complementary to the seed region of miR-221 (in red and orange) clearly separate from the background, which consists of the seeds of all the other miRNAs. For each plot the *x*-axis represents the sorted gene list from the most down-regulated (left) to the most up-regulated (right) mRNAs in miR-221 transfected cells and the *y*-axis shows the hypergeometric significance of each nucleotide word. The multiple testing bounds for adjusted *P* < 0.05 is: ±4.286 for 6-mers, ±4.199 for 7-mers, and ±3.923 for 8-mers.

Comparison of the average expression level of these probes between miR-221 vs. NC experimental groups was performed and each probe was then ordered according to fold change, from the most down-regulated to the most up-regulated of the miR-221-transfected cells group. The Sylamer algorithm, through the web-based interface Sylarray, was used to analyze the ordered gene list of mRNAs. First, the algorithm filters out genes with low complexity 3′UTRs and selects one probe for each of the maintained genes. Following this selection, Sylamer analysis was applied to a reduced list comprising 11,971 genes (Table S2). A highly significant (*p*−value =1 × 10^−15^) enrichment for miR-221 target sequences was detected in the 3′UTRs of many of the down-regulated genes (Figure 1).

According to Sylamer indications, the cut-off was chosen in correspondence to the peak closest to the start of the ranking in the 7-mer plot, which led to the selection of the first 1800 most down-regulated mRNAs in miR-221 transfected cells (reduced FC < −1.15), (Figure 1). Within this cut-off, the algorithm detected the presence of at least one seed complementary region for miR-221 in 602 genes (33.4%) (Table S3). All included the hexamer TGTAGC (corresponding to the nts 2–7 of miR-221 seed); 238 included the heptamer TGTAGCA (corresponding to the nts 2–8 of seed); 310 included the heptamer ATGTAGC (corresponding to the nts 1–7 of seed); 120 included the octamer ATGTAGCA (corresponding to the nts 1–8 of the seed).

To assess the value of these results, we verified the presence of validated targets within these lists. In a list of 35 published targets of miR-221 (http://www.genego.com/, August 2012), 10 (ARHI, ESR1, SLM1, KIT, PIK3KR1, SEMA6D, SLC4A4, TNF, FOG2, ICAM) could not be evaluated because their expression was undetectable in SNU-398 cells. Among the remaining 25 genes, 14 (PUMA, BMF, CDKN1B, CDKN1C, GARNL1, MDM2, THRB, APAF1, TIMP3, TRPS1, ZADH2, RP42, DVL2, and Connexin43) were identified by Sylamer within the list of the 1800 most down-regulated genes; five (SEC62, FOXO3, Hox-B6, SIP2, and PTPR-mu), although still down-regulated, were outside the selected cut-off and six were found slightly up-regulated (Dicer, BIM, NLK, PTEN, REDD1, and Ets1) (Table S4). We cannot exclude the possibility that down-regulation of these latter genes could be detectable at protein but not mRNA level. In any case, these results indicate that a significant portion (56%) of the published validated gene targets could have been identified through this approach and suggest that many of the additional targets yet to be validated are likely present within this list.

The published gene targets detected by Sylamer contained at least 1 or 2 complementary sites for the 7-mer seed within their 3′UTRs. As expected, with the exception of RALGAPA1 (or GARNL1), THRB and Connexin43, the remaining 10 proven gene targets were predicted by at least one of the online programs MicroCosm, Targetscan or Diana microT, indicating that presently known gene targets were largely identified through an initial scanning using available online predictions.

We intersected the 602 genes that emerged from Sylamer analysis with genes predicted by three online algorithms (MicroCosm, Targetscan, Diana microT). Overall, 125 genes identified by Sylamer (19.9%) were also predicted by online algorithms (Table S5). This set of 125 genes are therefore not only predicted by online algorithms, but also emerged in an experimental setting based on microarray expression analyses, thus adding a new level of confidence to these genes as miR-221 real targets.

### miR-221 induced pro-proliferative pathways involved in cancer

We investigated the biological processes associated with the genes that emerged from Sylamer analysis: the 602 genes with at least one site matching the seed sequence of miR-221 were analysed for detecting their association with molecular pathways through the use of Genespring GX 11 (Agilent Technologies) and GeneGO (Thomson Reuters) programs.

Both methods provide a rapid categorization of large lists of genes into functionally related groups of genes. At the *p*-value cut-off of 0.05, Genespring and GeneGO found several pathways that were enriched in genes targeted by miR-221 (Tables 1, S6). Many of these genes were related to cell cycle regulation and apoptosis.

**Table 1:** **Pathways affected by miR-221, based on Genespring and GeneGo analysis of target genes**.

**Pathway**	**Average *p*-values^a^**
	**GeneGo**	**Genespring**
Cell adhesion: ECM remodeling, Cadherin-mediated, Ephrin signaling	8,55E-05	2,93E-03
TGF, WNT, and cytoskeletal remodeling	1,71E-05	2,89E-02
P53 signaling pathway	1,33E-04	2,20E-02
Cell cycle regulation	9,94E-05	2,16E-02
Growth factors signaling	7,10E-05	2,35E-02
Signaling in macrophages, B and T lymphocytes	1,78E-04	2,82E-02

a *Based on data of Table S6*.

**Figure 2:**
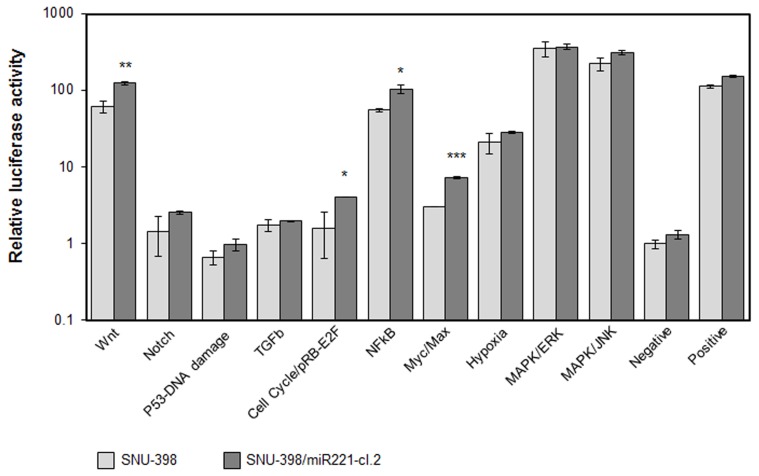
**Cellular pathways induced by miR-221.** The Cignal Finder 10-Pathway Reporter Arrays (SABiosciences) was employed. Relative luciferase activity was measured in SNU-398 (low miR-221 expression) or in SNU-398/miR221 clone 2, which shows a miR-221 expression about 2.5-fold higher than in SNU-398 cells (see Figure S1). Each condition was measured in triplicate. A *t*-test was used to compare the average expression of each condition in the two cell lines. Wnt, cell cycle, NFkB, and Myc exhibited a significant increased activity in the SNU-398/miR221 cells. ^*^p-value ≤ 0.05; ^**^p-value ≤ 0.01; ^***^p-value ≤ 0.001.

To directly investigate the involvement of miR-221 in cancer processes, we used Cignal Finder 10-Pathway Reporter Arrays kit (SABiosciences), a commercial reporter array that allows for simultaneous evaluation of 10 cancer-related signaling pathways activation levels in cells, using specific pathway-focused transcription factor-responsive firefly luciferase constructs. This kit was used to evaluate the ability of miR-221 to induce cancer-related pathways in the SNU-398/miR221 clone 2 cells, engineered to stably express increased levels of miR-221 (Figure S1). We found that four pathways, Myc/Max, NFkB, Wnt/β-catenin, and RB-E2F, were significantly induced in miR-221 expressing cells, compared to the original cells (*p*-value <0.05) (Figure 2), thereby confirming miR-221 involvement in cellular processes that can induce proliferation and suppress apoptosis.

### Validation of novel cancer-associated targets of miR-221

From the list of genes identified by Sylamer and the list of pathways significantly affected by miR-221, we focused our attention on genes involved in cell proliferation and apoptosis regulation to individually validate them as miR-221 targets. They included the cell cycle regulators retinoblastoma 1 (RB1) and WEE1, the pro-apoptotic gene Apoptotic Peptidase Activating Factor 1 (APAF1), CCCTC-binding factor (CTCF), a transcriptional repressor and Annexin A1 (ANXA1). In addition, we also investigated Fas Ligand (FASLG), which contains a miR-221 target region in its 3′UTR, but was not within the Sylamer-derived-602 genes list. The genes studied are listed in Table 2 and in Figure S2.

**Table 2:** **Genes individually validated as miR-221 targets**.

**Gene**	**Gene symbol**	**Sylamer position^a^**	**3′UTR length (bp)**	**Number of miR-221 target sequences**	**Fold-change^b^**	**Online prediction^c^**
Retinoblastoma 1	RB1	1087	1915	2	−1, 2	
Apoptotic Peptidase Activating Factor 1	APAF1	430	2945	2	−1, 3	
Annexin A1	ANXA1	1580	718	2	−1, 16	M
WEE1 homolog (*S. Pombe*)	WEE1	1598	1382	1	−1, 16	
CCCTC-binding factor (zinc finger protein)	CTCF	302	1308	2	−1, 37	D
Fas ligand (TNF superfamily, member 6)	FASLG	N/A	907	1	N/A	

a *Position within the first 1800 most down-regulated mRNAs*.

b *Calculated on the basis of microarray experiments (3 miR-221 vs. 4 NC2)*.

c *M, Microcosm; D, Diana microT; T, Targetscan*.

**Figure 3:**
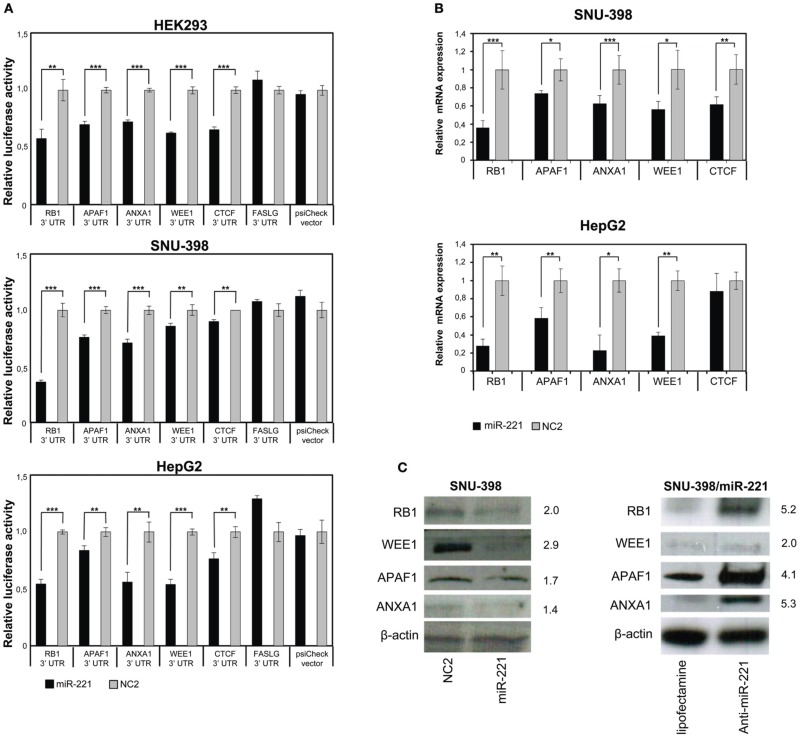
**Validation of few individual miR-221 target genes. (A)** Luciferase assays were performed in Hek293, SNU-398, and HepG2 cells transfected with miR-221 or negative control (NC2), together with psiCheck luciferase vectors containing portions of the 3′UTR of the investigated genes placed downstream the renilla luciferase gene. The renilla luciferase activity was normalized on firefly luciferase activity. Compared to NC2 controls, miR-221 induced a significant decrease in luciferase activity of the vectors containing the RB1, APAF1, ANXA1, WEE1, and CTCF 3′UTR. In contrast, no change in luciferase activity could be detected for the FASLG 3′UTR-luciferase vector and the psiCheck control vector. ^**^p-value ≤ 0.01; ^***^p-value ≤ 0.001. **(B)** RB1, APAF1, ANXA1, WEE1, and CTCF mRNA expression was measured in SNU-398 and HepG2 cells following transfection with miR-221 or NC2 using Real Time PCR. A significant reduction in mRNA levels was detected for all the investigated genes, with the exception of CTCF mRNA in HepG2 cells. ^*^p-value ≤ 0.05; ^**^p-value ≤ 0.01; ^***^p-value ≤ 0.001. **(C)** Protein expression levels were assessed by Western blot analysis. miR-221 induced a decrease in RB1, APAF1, ANXA1, and WEE1 proteins in SNU398 cells, while transfection of anti-miR-221 into SNU-398/miR-221 stable clone induced an increase in target protein levels. Numbers indicate the fold change decrease or increase of proteins expression in miR-221 or anti-miR-221 transfected cells vs. the respective controls. Protein expression levels were normalized vs. β-actin expression.

To validate potential miR-221 target genes, we first performed luciferase assays. We cloned a portion of the 3′UTRs containing miR-221 target sequences into a psiCheck-2 reporter vector, downstream the luciferase reporter gene. We assayed the luciferase activity in the presence or absence of added miR-221 mimics in three different cell lines: HEK-293 (embryonic kidney derived cells) and two hepatocarcinoma derived cells (SNU-398, HepG2). The reporter vector constructs were transfected into cells together with miR-221 precursor or negative control (NC2). We found that miR-221 induced a significant decrease in luciferase activity of all the vectors containing 3′UTRs of genes identified through the Sylamer approach. Instead, no change in luciferase activity could be detected for the psiCheck-FASLG 3′UTR vector (Figure 3A). In the case of RB1, to confirm that the decrease in luciferase activity was specifically linked to the miR-221 complementary regions present in the 3′UTR, we prepared a mutant form of psiCheck-RB1 3′UTR (psiCheck-RB1 3′UTR mutated) that lacks the miR-221 target sequences within the RB1 3′UTR. Mutation of these sites made the vector unresponsive to miR-221 inhibitory effect (Figure S3).

The ability of miR-221 to induce a decrease in RB1, APAF1, ANXA1, WEE1, and CTCF expression was further demonstrated by measuring the relative amount of their mRNAs in SNU-398 and HepG2 cell lines. We found a significant decrease (*p*-value <0.003) of RB1 mRNAs in both cell lines transfected with miR-221 (−64% in SNU-398 and −72% in HepG2); similarly, we found 26 and 42% decrease in APAF1 mRNA level, respectively, in SNU-398 and HepG2 (*p*-value <0.015); miR-221 caused a decrease of 38% (SNU-398) and 77% (HepG2) of ANXA1 mRNA (*p*-value <0.018); WEE1 mRNA was 44% (SNU-398) and 61% (HepG2) less expressed in presence of microRNA (*p*-value <0.013) and CTCF mRNA showed a 38% decrease in miR-221 transfected SNU-398 cells (*p*-value = 0.006), while in HepG2 we found a slight (but not significant) decrease (Figure 3B), meaning that specific cell background could modulate microRNA effects on target genes.

Finally, protein expression analyses revealed that miR-221 could indeed decrease the levels of RB1, WEE1, APAF1, and ANXA1 proteins in SNU-398 cells; conversely, an oligonucleotide anti-miR-221 induced their increase in SNU-398/miR-221 stable clone (Figure 3C). We could not test CTCF protein, because available antibodies produced poor quality results.

## Discussion

We have added a number of novel gene targets to those already identified for miR-221. In this study, we did not use the traditional “*in silico*” prediction, followed by the individual validation of the most interesting ones. In fact, through this approach, various important miR-221 gene targets have been already identified (Galardi et al., 2007; le Sage et al., 2007; Fornari et al., 2008; Medina et al., 2008; Garofalo et al., 2009; Gramantieri et al., 2009; Zhang et al., 2010a,b; Quintavalle et al., 2012) (see also Table S4). Here, we used an approach that was based on Sylamer analysis of gene expression profiling. Sylamer couples experimental gene expression results to the search for microRNA target sequences in the 3′UTR of genes. By using this approach, we could identify 602 miR-221 gene targets, among which 125 were also predicted by at least one prediction algorithm. For this latter group of genes, our experimental results add a new level of confidence to their inclusion among the true targets of miR-221.

The enforced expression of miR-221 did not alter the level of miR-222. Hence, the targets reported by this study may actually be specifically regulated just by miR-221. However, by sharing the same seed sequence, it is possible that many of the discovered genes could potentially be targets of miR-222 too. More specific experiments would be required to formally prove this point.

To achieve these results, in this study we did not employ the traditional *t*-test analysis, which is used to select the genes with a significant differential expression level between two groups of samples. In fact, this approach would have been too stringent and many real targets excluded. For example, two known targets, such as CDKN1B (Gramantieri et al., 2008) and BMF (Gramantieri et al., 2009) were among the down-modulated genes, but their *p*-value was not significant (Table S2). Analysis based on Sylamer did not produce this bias. Even slight, apparently non-significant effects on mRNA modulation could be picked up by Sylamer if the gene is present in the context of a group of genes that share the characteristic of having homology with the same miRNA's seed. It should also be considered that this assay takes into account only miRNA effects on the stability of target mRNAs, without considering its effect on mRNA translation, which is often predominant in mammalian cells.

To validate some of the results, we also applied the traditional approach to few of them, RB1, APAF1, WEE1, CTCF, and ANXA1: the assessment of mRNA and protein levels following miRNA or anti-miRNA enforced expression and luciferase assays were used to prove direct interaction with predicted target sites. In addition, for RB1, mutation of the putative miR-221 target site was shown to abolish the miR-221 regulation. Notably, we also identified Anxa1 as strongly down-regulated in a proteomic screening performed in SNU-398 cells transfected with miR-221 (Corrales, data not shown), further confirming Anxa1 as target of miR-221 regulation. APAF1 was recently independently demonstrated as miR-221 target in lung cancer derived-cell lines (Quintavalle et al., 2012), thereby giving support to our finding. Conversely, FAS ligand, a gene that contains a miR-221 targeting site within its 3′UTR, but was not present among the down-regulated mRNAs detected in this study, was not validated as a miR-221 target by luciferase assay. In general, all the individually tested genes confirmed to be targets of miR-221, suggesting that most, if not all the 602 genes identified through expression profiling and Sylamer analysis may represent truly novel gene targets modulated by miR-221. Taken together, these findings show that this approach represents a feasible and effective method for the identification of large sets of valid miRNA targets.

Pathway analysis of the 602 putative miR-221 target genes revealed that these genes are involved in cellular processes related to cell cycle regulation and apoptosis. Experimental tests revealed that various pathways, which included WNT/β-catenin, E2F/RB cell cycle, MYC and NFkB, may be promoted by miR-221 over-expression.

Previous experiments demonstrated that miR-221 was able to induce tumor cells proliferation, both *in vitro* (Galardi et al., 2007; le Sage et al., 2007; Fornari et al., 2008; Gramantieri et al., 2009) and *in vivo* models (Pineau et al., 2010; Callegari et al., 2012). Among these pathways, inhibition of RB1 protein may explain the activation of the E2F pathway and support the important role of miR-221 in cell cycle progression. Indeed, it was previously shown that miR-221 can repress the CDK inhibitors CDKN1B and CDKN1C (both confirmed in this study), and RB1 represents the third negative regulator of cell cycle that appear to be controlled by miR-221. This finding supports the observation that miR-221 could induce a significant increase of Hep3B HCC cells in S-phase and a decrease of cells in G1-phase (Fornari et al., 2008). This finding suggests that impairing cell cycle control mechanisms is one of the main effects of miR-221 over-expression in human cancer. The present work has led to the discovery of hundreds of new miR-221 target genes and provides an example of the multiple molecular functions that a single deregulated miRNA could affect. This is relevant for the establishment of miRNA-based therapeutics: multiple pathways could be simultaneously affected when miRNA or anti-miRNA are given as therapeutic molecules. The tumor promoting activity of miR-221 has been recently confirmed and supported by *in vivo* models also by showing the anti-tumor effect achieved by anti-miR-221 oligonucleotides: anti-miR-221 could significantly inhibit tumor cell proliferation of human HCC xenografts (Park et al., 2011) as well as reduce the number and size of liver tumors in a transgenic mouse model (Callegari et al., 2012). The present results add new information about the molecular pathways controlled by miR-221, thus contributing to explain how this microRNA could promote tumorigenesis and how to better assess the effect of its inhibition.

This work, by showing that the Sylamer algorithm could reveal the enrichment for a miRNA seed sequence among a group of down-regulated mRNAs, represents a general approach that could be applied to any miRNA for the experimental identification of a wide range of gene targets. In the case of miR-221, the list of modulated mRNAs was used to improve our understanding of its role in tumor pathways. However, these results may potentially be useful for defining the molecular mechanisms that are influenced in any other physiological or pathological condition in which miR-221 could be eventually implicated.

### Conflict of interest statement

The authors declare that the research was conducted in the absence of any commercial or financial relationships that could be construed as a potential conflict of interest.

## Supplementary material

Table S1 **Primers for amplification of 3′UTRs**.

Table S2 **List and ranking of genes analyzed by Sylamer algorithm**.

Table S3 **Targets with at least 1 complementary region to miR-221 seed**.

Table S4 **Previously validated miR-221 target genes in Sylamer analysis**.

Table S5 **miR-221 targets from Sylamer analysis and their prediction by three online algorithms**.

Table S6 **Significant pathways affected by miR-221 target genes based on Genespring and GeneGo analysis**.

Figure S1 **Expression levels of miR-221 and miR-222 in transient and stable cell clone**.

Figure S2 **miR-221 target sites within the 3′UTRs of the human genes RB1, APAF1, WEE1, ANXA1, CTCF, and FASLG**.

Figure S3 **Mutant 3′UTR of the RB1 gene becomes insensitive to miR-221 regulation**.
