# Supplementary material for: miR-221 affects multiple cancer pathways by modulating the level of hundreds messenger RNAs
Source: Front Genet. 2013 Apr 25;4:64. doi: 10.3389/fgene.2013.00064 (PMC3635019; doi:10.3389/fgene.2013.00064)
Supplement: Figure S1 — Expression levels of miR-221 and miR-222 in transient and stable cell clone. [file Presentation1.PDF]

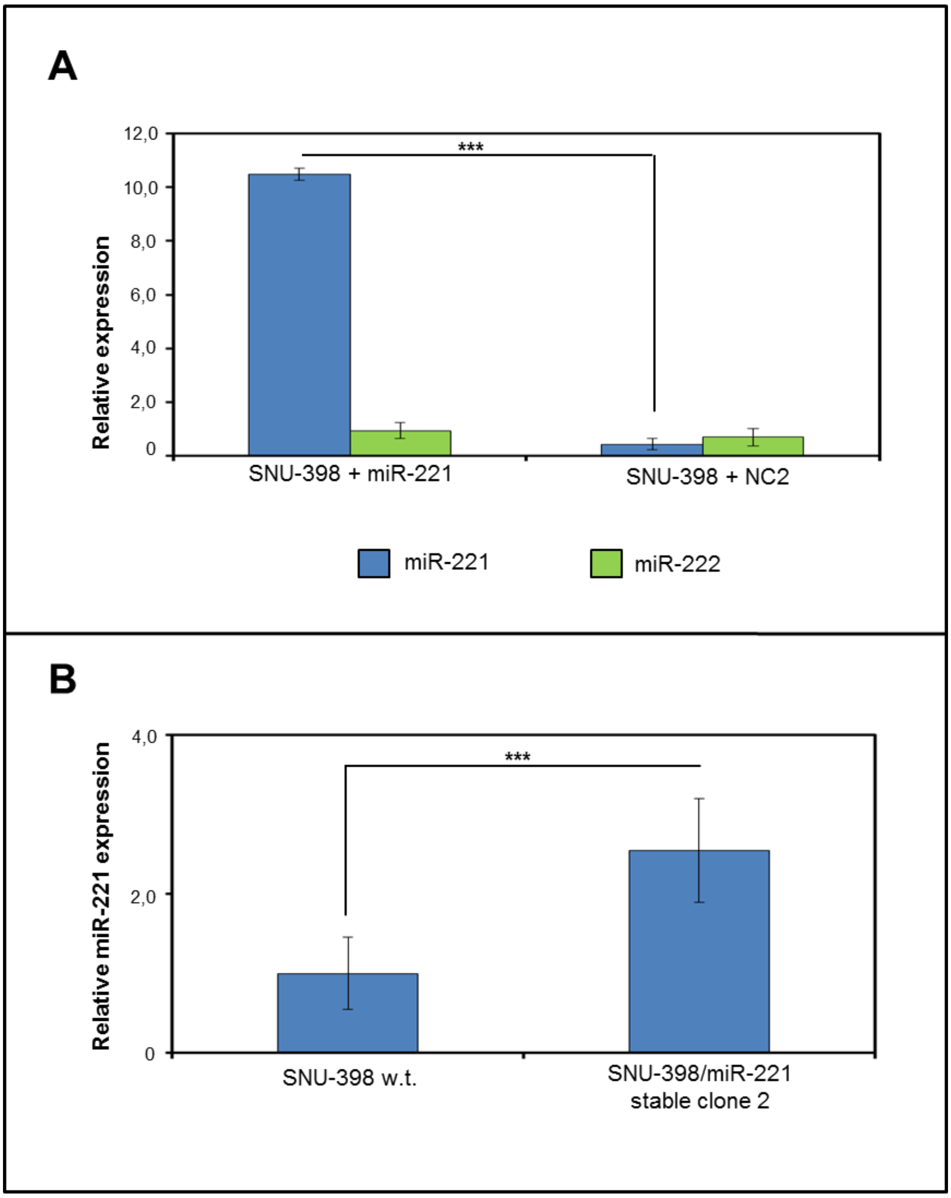

Supplementary Figure 1. *Expression levels of miR-221 and miR-222 in transient and stable cell clone.* **A.** Transient transfection of miR-221 precursor in SNU-398 ("SNU-398 + miR-221") induced a ≈ 10-fold induction in miR-221 expression, compared to NC2 transfected cells ("SNU-398+NC2"). No changes in miR-222 expression were found in the same samples. **B.** For miR-221-expressing stable cell clones, SNU-398 cells were transfected with pMIF-GFP-miR-221 vector and cells selected by zeocin. Among the selected cell clones, clone 2 exhibited a 2.5-fold increase in miR-221 expression in comparison with SNU-398 wild type cells ("SNU-398 w.t."). * = p-value ≤ 0,05; ** = p-value ≤ 0,01; *** = p-value ≤ 0,001.
