# Supplementary material for: miR-221 affects multiple cancer pathways by modulating the level of hundreds messenger RNAs
Source: Front Genet. 2013 Apr 25;4:64. doi: 10.3389/fgene.2013.00064 (PMC3635019; doi:10.3389/fgene.2013.00064)
Supplement: Figure S2 — miR-221 target sites within the 3′UTRs of the human genes RB1, APAF1, WEE1, ANXA1, CTCF, and FASLG. [file Presentation2.PDF]

RB1 3’utr (nucleotide 236): 5’.. UGCCAUUUAAAAAGU**UGUAGC**AG.. 3’
| | | ||||||
Hsa-miR-221: 3’ CUUUGGGUCGUCUGUU**ACAUCG**A 5’

RB1 3’utr (nucleotide 351): 5’.. UACUUUGCCUUCUUU**UGUAGC**AU.. 3’
| | ||||||
Hsa-miR-221: 3’ CUUUGGGUCGUCUGUU**ACAUCG**A 5’

APAF1 3’utr (nucleotide 1): 5’..AAUAGUUAAGCAUU**AAUGUAG**U.. 3’
| | | |||||||
Hsa-miR-221: 3’ CUUUGGGUCGUCUG**UUACAUC**GA 5’

APAF1 3’utr (nucleotide 163): 5’..GGAUGAAUAAUAUU**AAUGUAGC**UU.. 3’
| | ||||||||
Hsa-miR-221: 3’ CUUUGGGUCGUCUG**UUACAUCG**A 5’

ANXA1 3’utr (nucleotide 100): 5’..CUUCAACAGGAUUACAG**UGUAGCU**A.. 3’
| | | ||| |||||||
Hsa-miR-221: 3’ CUUUGGGUCGUCUGUU**ACAUCGA** 5’

ANXA1 3’utr (nucleotide 254): 5’..CAUGAGAAAGAUGUCU**AUGUAGCU**G.. 3’
| || | | ||||||||
Hsa-miR-221: 3’ CUUUGGGUCGUCUGU**UACAUCGA** 5’

WEE1 3’utr (nucleotide 562): 5’..GTCTTTGCTGTAAACT**TGTAGC**AT.. 3’
| | ||||||
Hsa-miR-221: 3’ CUUUGGGUCGUCUGUU**ACAUCG**A 5’

CTCF 3’utr (nucleotide 686): 5’.. AATTGGATCACAATC**ATGTAGC**AG.. 3’
| | |||||||
Hsa-miR-221: 3’ CUUUGGGUCGUCUGU**UACAUCG**A 5’

CTCF 3’utr (nucleotide 838): 5’.. TTTTCTTTGCCCTGTT**TGTAGCT**G.. 3’
| || |||||||
Hsa-miR-221: 3’ CUUUGGGUCGUCUGUU**ACAUCGA** 5’

FASLG 3’utr (nucleotide 155): 5’..CACAGGGTTCAAAATGTC**TGTAGCT**.. 3’
| | | | | |||||||
Hsa-miR-221: 3’ CUUUGGGUCG-UCU-GUU**ACAUCGA** 5’

**Supplementary Figure 2. *miR-221 target sites within the 3’UTRs of the human genes RB1, APAF1, WEE1, ANXA1, CTCF and FASLG*.** miR-221 seed sequence and mRNA complementary regions are highlighted in bold letters. The position of miR-221 seed complementary region within the 3’UTR of each mRNA is in brackets.
