# Supplementary material for: miR-221 affects multiple cancer pathways by modulating the level of hundreds messenger RNAs
Source: Front Genet. 2013 Apr 25;4:64. doi: 10.3389/fgene.2013.00064 (PMC3635019; doi:10.3389/fgene.2013.00064)
Supplement: Figure S3 — Mutant 3′UTR of the RB1 gene becomes insensitive to miR-221 regulation. [file Presentation3.PDF]

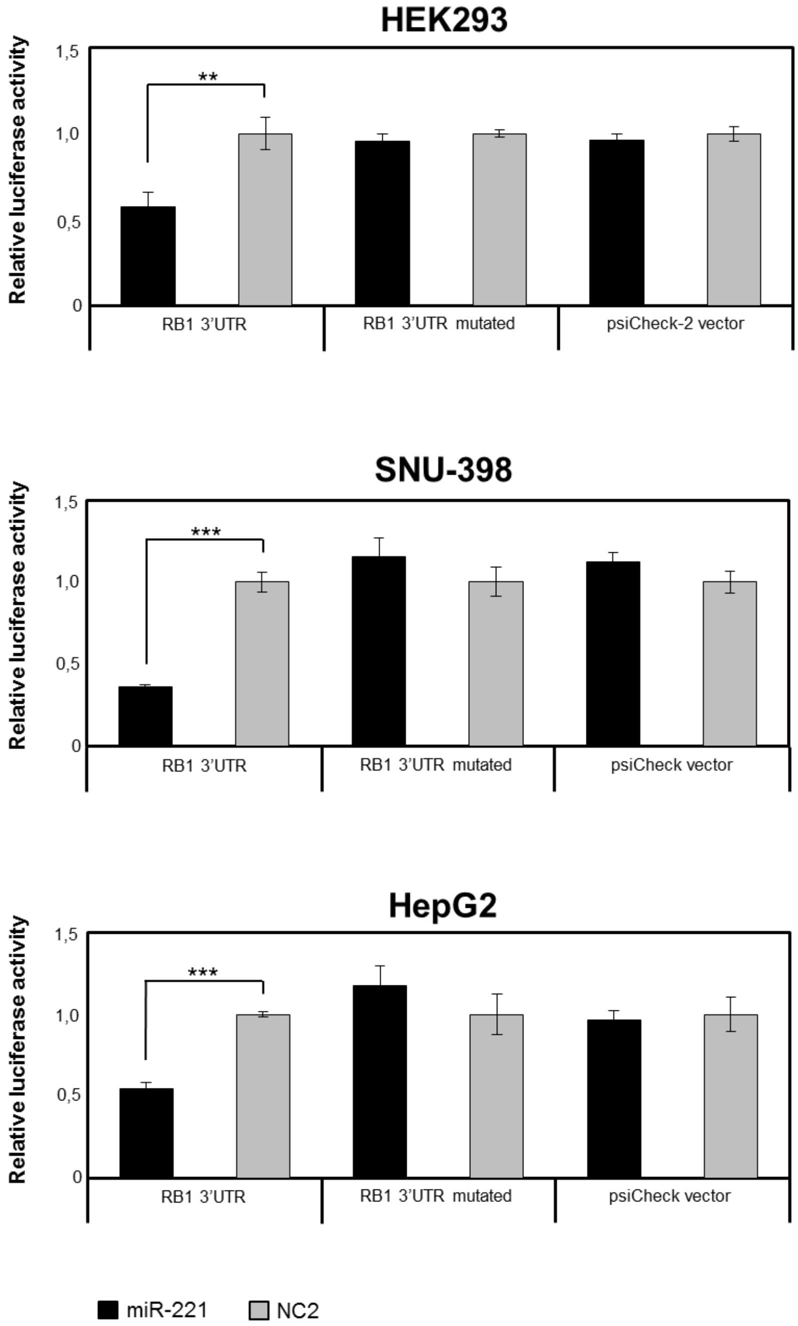

Supplementary Figure 3. ***Mutant 3'UTR of the RB1 gene becomes insensitive to miR-221 regulation.*** Luciferase assays were performed in Hek293, SNU-398 and HepG2 cells, transfected with psiCheck-RB1-3'UTR or psiCheck-RB1 3'UTR mutated constructs, in presence of miR-221 or negative control (NC2). Renilla luciferase activity was normalized on firefly luciferase activity. We found a significant decrease in relative luciferase activity in miR-221-transfected samples, compared to control, in all cell lines. In contrast, we did not find any change in luciferase activity in cells transfected with a mutated form of psiCheck-RB1 (psiCheck-RB1 3'UTR mutated), missing two miR-221 seed complementary regions in RB1 3'UTR. * = p-value ≤ 0,05; ** = p-value ≤ 0,01; *** = p-value ≤ 0,001.
